# Activities of daily living in older community-dwelling persons: a systematic review of psychometric properties of instruments

**DOI:** 10.1007/s40520-018-1034-6

**Published:** 2018-09-06

**Authors:** Marijke Hopman-Rock, Helmi van Hirtum, Paul de Vreede, Ellen Freiberger

**Affiliations:** 1 Research center Body@Work TNO (Netherlands Organization for Applied Scientific Research) and VU University Medical Center, Van der Boechhorststraat 7, 1081 BT Leiden/Amsterdam, The Netherlands; 2 Department of Public Health, Erasmus MC, University Medical Center Rotterdam, Rotterdam, The Netherlands; 3 Institute for Biomedicine of Aging, Friedrich-Alexander University Erlangen-Nürnberg, Kobergerstr. 60, 90408 Nuremberg, Germany; 4 Present Address: Sint Antonius Hospital, Koekoekslaan 1, 3435 CM Nieuwegein, The Netherlands; 5 Present Address: Concreet Onderzoeken and Toepassen, Hofzicht 2, 2641 LT Pijnacker, The Netherlands

**Keywords:** Aging, Function, Health status, Community dwelling, Activities of daily living, Assessment

## Abstract

**Background:**

Activities of daily living (ADL) are often used as predictors of health and function in older persons. This systematic review is part of a series initiated by the European Network for Action on Ageing and Physical Activity (EUNAAPA).

**Aim:**

To assess psychometric properties of ADL instruments for use in older populations.

**Methods:**

Electronic databases (Medline, EMBASE, AMED, Psycinfo, CINAHL) were searched, using MeSH terms and relevant keywords. Studies, published in English, were included if they evaluated one or more psychometric properties of ADL instruments in community-dwelling older persons aged 60 years and older. Combination scales with IADL were excluded. This systematic review adhered to a pre-specified protocol regarding reliability, validity, and responsiveness.

**Results:**

In total, 140 articles describing more than 50 different ADL instruments were included. Ten instruments which were applied in minimally three different articles of good quality (clear descriptions and adequate design according to the protocol), were evaluated for reliability, validity and responsiveness; each received a summary score. The four instruments with the highest scores were the Functional Autonomy Measurement System (SMAF), 5-items Katz list (although content and wording are often inconsistent across studies), Functional Independence and Difficulty Scale (FIDS) and the Barthel Index.

**Discussion:**

Critical reflection is essential to avoid unnecessary modifications and use of instruments that have not been documented to be valid or reliable.

**Conclusion:**

Based on this systematic review, we recommend the SMAF, 5-item Katz, FIDS and Barthel index as ADL measures for research and care practice in older populations.

**Electronic supplementary material:**

The online version of this article (10.1007/s40520-018-1034-6) contains supplementary material, which is available to authorized users.

## Introduction

In community-dwelling, older persons screening and assessing the ability to conduct activities of daily living (ADL), such as getting out of bed, toileting, bathing, dressing, grooming, and eating are frequently used. These measures are applied to detect early onset of disability and are key factors for care management [1] (note: key references are cited in text, see Appendix 1 for additional references). In most cases, this information is obtained with questionnaires and commonly used to refer to basic or personal ADL [i.e., self-care activities (B)ADL].

Few relevant studies, particularly as it relates to psychometric properties, exist in this area. One review by Fieo et al. [2], evaluate studies on ADL and IADL (Instrumental Activities of Daily Living) scales employing a measurement technique (item response theory; IRT) in adults over 60 years of age. These authors identify 12 different articles describing IRT analyses on combined ADL/IADL scales. The findings suggest that some IRT modified instruments were more sensitive in detecting preclinical stages of functional decline. Because of the limited number of identified studies (the use of IRT analyses is not common), the authors did not propose best practice recommendations for the use of ADL instruments. Therefore, further evaluation of existing ADL instruments is necessary to provide recommendations for researchers and clinicians.

The oldest and well-known ADL questionnaire is the list developed by Katz [3] in 1963. Since that point, several modifications have been proposed and other measurements developed and used to measure ADL and predict disability in older adults. Compared to functional performance tests (for a review see Freiberger et al. [4]), ADL measurement, in question format only, generally has weak reliability, validity, reproducibility and sensitivity to change [2, 5]. Further, as older community-dwelling adults are living independently, one could expect a prominent ceiling effect in these measures of basic functions. Nevertheless, a variety of such ADL instruments is used as a routine in studies in older adults. Most of them are documented and tested on validity and reliability [2A]. However, a lot of these instruments are not specially designed for use in community-dwelling older populations, and the question remains how valid and reliable they are when used in such a context.

To our knowledge, no information on psychometric properties comparing existing questionnaires on ADL functioning in older community dwelling persons is available. Therefore, the aim of this study was to conduct a systematic review on the psychometric properties of ADL measurements that are currently used in research on older community-dwelling populations. A second aim was, to provide recommendations in practice for researchers, clinicians, and healthcare professionals. This systematic review is part of a series of reviews initiated by the European Network for Action on Ageing and Physical Activity (EUNAAPA http://www.eunaapa.org [4, 6–8].

## Methods

We adhered to a pre-specified protocol regarding search strategy and inclusion and exclusion criteria by the EUNAAPA review group based on a checklist for among others reliability, validity, and responsiveness and pre-specified definitions of subtypes of validity and quality of a study (see Table 1 [9]).

**Table 1:** Quality criteria for properties of ADL instruments (Table source: Terwee et al. [9])

Property	Definition	Quality criteria^a,b^
Content validity	The extent to which the domain of interest is comprehensively sampled by the items in the instruments	+: Positive rating −: Poor rating ?: Moderate rating
Predictive validity	The extent to which the instrument had the ability to predict onset of difficulties in functioning or negative health outcomes over time (e.g., mortality)	+: High scores with regard to methods, design and results (OR or AUC) ?: Doubtful design or method (e.g., small sample size) −: Inappropriate methods or lack of significant results
Construct validity^c^	The ability to discriminate between subgroups e.g., age groups, gender	+: High scores with regard to methods, design and results (clear group definitions and significant results) ?: Doubtful design or method (e.g., small sample size) −: Inappropriate methods or lack of significant results
Concurrent validity	Established by simultaneously applying a previously validated tool or test, and comparing the results	+: Comparison to other instrument with significant results (*r* > 0.80) ?: Doubtful design or method (e.g., small sample size); significant but small results *r* > 0.60–0.80 −: Inappropriate methods or lack of significant results
Reliability^d^	An indicator of the consistency of a measurement in terms of internal consistency with stability over time (reproducibility) and the degree of which the measurement is free of measurement error (internal consistency)	+: (good) Intraclass Correlation Coefficient (ICC) or Kappa > 0.70 ?: (moderate) CC 0.70 − 0.60 or *r* > 0.80 −: (poor) ICC or Kappa < 0.70, despite adequate design and method
Responsiveness	The instrument’s ability to detect important change over time in the concept being measured, and may be defined as the extent to which a method detects minimal clinically relevant change over time	+: A power calculation for sample size presented adequate design and sufficiently described ?: Doubtful design or method (e.g., no hypotheses)
Floor- and ceiling effects	The number of respondents who achieved the lowest or highest possible score	+: ≤ 15% of the respondents achieved the highest or lowest possible scores ?: Doubtful design or method −: > 15% of the respondents achieved the highest or lowest possible scores, despite adequate design and methods
Overall quality of individual study	The degree to which one can assign qualitative meaning to quantitative scores	+: Clear description of study population, adequate description of instrument, adequate design for evaluating psychometric properties ?: Doubtful description of either study population, or instrument but with reference given or method −: Poor description of study population, OR instrument and no reference given, and poor method

*AUC* area under the curve; *ICC* intraclass correlation coefficient; *OR* odds ratio

^a^ + = Positive rating; − = poor rating; ? = moderate rating

^b^Doubtful design or method = lacking of a clear description of the design or methods of the study, sample size smaller than 30 subjects (e.g., subgroup analysis), or any important methodological weakness in the design or execution of the study

^c^Convergent and discriminant validity are usually both considered subcategories or subtypes of construct validity

^d^We added Cronbach’s alpha as a measure of internal consistency: 0.00 to 0.69 = poor; 0.70 to 0.79 = fair ; 0.80 to 0.89 = good ; 0.90 to 0.99 = excellent/strong

### Search strategy

Electronic databases (Medline, EMBASE, AMED, Psychinfo, CINAHL) were searched from their inception to August 2012 and updated in November 2016. Using MeSH terms and relevant keywords, six semantic categories were entered: “ADL, Questionnaire, Age (60 years and older), Setting (community dwelling), Reproducibility, Validity”. Reference lists of review articles and included papers were scanned to identify further potential studies. The search was restricted to English language and peer-reviewed journal articles (see Appendix 2 for a comprehensive overview).

### Eligibility and selection criteria

To be included a study had to meet the following five criteria: (1) investigate at least one of the mentioned psychometric properties of an ADL instrument [reliability, validity, reproducibility and sensitivity to change]; (2) measure (B)ADL in a separate (sub)scale; (3) include a population 60 years of age or older or with a mean age above 65 years or separately reported on this age group; (4) address community-dwelling older persons, and (5) have a sample size of at least 30 participants.

Studies were excluded if they did not utilize a separate ADL scale; if the instrument used was developed for populations with specific diseases; or if the ADL scale had less than three items; or was rated inadequate for reporting reliability, validity, and/or responsiveness.

### Data extraction and evaluation of psychometric evidence

Five independent reviewers performed abstract scanning, selection of full-text articles, and data extraction. Disagreements that could not be solved by discussion between two reviewers were judged by one of the other reviewers. Full-text papers were obtained if the inclusion criteria could be clearly determined from the abstract or eligibility was not sure. In the case where further information was needed authors of articles were contacted. The five independent reviewers read the full text articles and rated them as eligible or not.

The overall quality of eligible studies was based on three domains (study population, adequate description of instrument, adequate design for evaluating psychometric property); [9] and rated as good (+), poor (−), or moderate (?) (Table 1). Thus, a clear description of the study sample, the measurement and design for evaluating psychometric properties was required to receive a positive rating (+). If the authors failed providing a clear description on one domain, it was rated moderate (?) and when it failed on two or more domains it was rated poor (−). It should be understood that a good quality article contained information about psychometric properties that were in the next stage graded as positive, moderate or negative (for instance the reliability or validity was well described but was not good enough).

To evaluate the strength of evidence for the psychometric properties of instruments, the domains ‘Quality’, and ‘Quantity’ were used. Quality of the evidence could be positive or negative for individual studies and was rated by the reviewers according to the checklist as presented in Table 1 [9, 10]. The Quantity was defined as the number of studies. Based on all quality ratings of the description of psychometric properties by the reviewers, the eligible articles were given an overall rating (+, ++, or −) by the first author (average ratings of the reviewers). Instruments with minimally three positively rated articles were included for further evaluation. This was an ad hoc criterion see Appendix 3. Articles were listed by first author, abstract number and year of publication. More recent articles (updated) display the name of the first author followed by the year of the online publication.

Finally, the evaluation of the psychometric properties found in quality articles were summarized in Table 2. Further discussions of the top-rated instruments are described later in the text.

Studies including ADL instruments report predictive, discriminant, construct and concurrent validity (see Table 1). Therefore, the current review will examine these four types of validity and reliability and responsiveness (including ceiling effects).

## Results

**Fig. 1:**
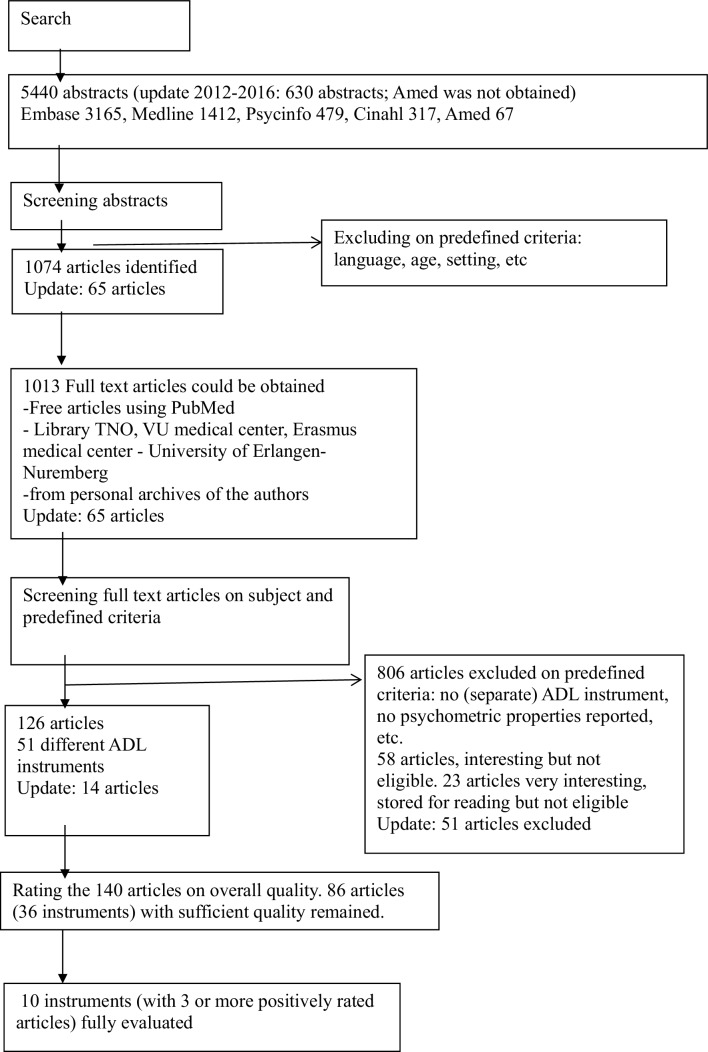
Flow chart of the selection process

The literature search identified 6070 abstracts (5440 + 630 in the update 2016). After screening the abstracts (abstract lists with full references available on request), 1139 full papers (including 65 articles in the update) were identified and 1078 articles obtained and further screened for inclusion or exclusion (Dropbox was used as a filesharing database). The flow of the selection process can be viewed in Fig. 1.

In total, 140 (including 14 articles in the update) articles describing 51 different ADL instruments and modified versions were included and overall quality further evaluated (see Excel worksheet object in Appendix 3). In the data extraction process, 54 studies investigating 34 different instruments were excluded, due to poor overall quality of the article (see Appendix 3). 86 articles remained, describing 36 different instruments. Instruments with three or more positively rated articles from this list [see Appendix (*N* = 56) [11–17, 18, 19–22, 23, 24–46, 47, 48–59, 60, 61–66]] were included for further evaluation (see Table 2). The results for reliability, validity, and responsiveness including ceiling effects were summarized and reported in Table 2 (sorted by number of positively rated articles on the ADL instrument). Two articles explicitly included ceiling effects. The data from La Plante [47], for two items (eating and bathing) reveal a 97% independence for eating (age 65–74) and 66% for bathing (age 85+). This data suggests that eating is not a good robust indicator of independence due to ceiling effects, whereas bathing is a much more sensitive indicator of independence. Saito [65] reported minimal ceiling effects in the FIDS compared to the Barthel.

**Table 2:** Summary of reviewed outcome measure’s properties (scores summarized by first author, best instrument outcomes reported in text)

Instrument (described in Refs.)	Ordered by number of positively rated (quality) articles *N* = 56	Reliability	Validity	Responsiveness
Katz 6 items [11–17,18,19–20]	10	+?	?	?
OARS (Older Americans Resources and Services) ADL scale [21–22,23,24–27]	7	−	?	−
Barthel Index^a^ [28–34]	7	+?	?	++
Katz 7 items [35–41]	7	+	?	+?
Katz 5 items [42–46,47,48]	7	+	+	+?
SMAF^b^ functional autonomy measurement system [49–54]	6	++	++	++
Katz unspecified [55–57]	3	+	−	−
NHIS ADL^c^ National health Interview Survey [58–59, 60]	3	+?	+	−
FIM (functional independence measure) [61–63] FIM phone	2	+ (Motor component)	?	+
FIM observation	1	+	−	−
FIDS (Functional Independence and Difficulty Scale) [64–66]	3	+	+?	+

+ = positive rating; ? = moderate rating or do not know ; − = poor rating

^a^Barthel Index Phone version failed to measure reliable in moderate and severe disabled patients

^b^SMAF clinical version is the most valid

^c^Qualitative study (Jobe et al. [60]) revealed problems with interpreting the questions

Four instruments with a minimum rating of three times a ‘plus’ in Table 2 are described hereunder.

The SMAF (Functional Autonomy Measurement System) was the only instrument with very good ratings in all three domains (validity, reliability and responsiveness). The SMAF is a 29-item scale based on the World Health Organization classification of disabilities. It measures functional ability in five areas: activities of daily living (ADL) (7 items: eating, washing, dressing, grooming, urinary continence, fecal continence and using the bathroom), mobility (6 items), communication (3 items), mental functions (5 items), and instrumental activities of daily living (IADL) (8 items). Each item is scored on a 4-point scale from 0 (independent) to 3 (dependent) for a maximum score of 87. For every item that has a rating of 1 or higher (i.e., not independent), the human resources (help or supervision) required to support the level of disability in this specific area and the stability of these resources were evaluated. The SMAF must be administered by a trained health professional (time approximately 40 min in total), who scores the individual after obtaining the information by questioning the subject and proxies or by observing [67]. A test–retest analysis of the ADL scale in the clinical version revealed a Cohen’s Kappa of 0.74, inter-rater reliability Cohen’s kappa of 0.81 and ICC of 0.96 (CI 0.92–0.97) [56]. Inter-rater reliability in another study showed 72.7% agreement (weighted κ 0.66) and test–retest 76% agreement, with weighted kappa 0.81. Discriminant validity was also measured as the correlation with nursing time for care and was 0.88 [52, 55, 57]. Differences between care client groups were significant (*p* < 0.01) revealing a good construct validity and responsiveness [53]. The results for the ADL scale in the telephone survey questionnaire were not as good revealing an ICC of 0.73 (CI 0.48–0.84) [51] Hebert and colleagues warn that “a survey method (note: they used a telephone survey) is not valid for generating accurate estimates of disabilities to plan services or determine budget requirements for responding to the needs of a population”.

The 5 item Katz list has reasonably good reliability, validity and responsiveness on average as reported in seven articles. However, the content of the five items could differ; two articles reported using “grooming” instead of “transferring”. Also, the wording for inventory items were not consistent. For example, we found eating to be described by both eating and feeding and toileting to be described as toileting or using the toilet. The inconsistent terminology also occurred for the Katz 6 and 7 item versions and the unspecified version of the Katz. Besides the items, also the wording of answer categories could be different (or was not mentioned). Kosloski et al. [48] reported a test–retest coefficient of 0.82 (*p* < 0.05) showing a reasonable reliability. Covinsky et al. [42] found that “the number of ADL reported 2 weeks before hospital admission was significantly associated with mortality 1 year later”. This data also supports a reasonable predictive validity. LaPlante [47] reported “Importantly, the ADL items are increasingly Guttman scalable with age, with CS = 0.77 (CS = 0.64 excluding extreme values) at ages 18–34 years rising to CS = 0.93 (CS = 0.82 excluding extremes) at ages 85 years and older, approaching near-perfect Guttman scalability.” (*CS* coefficient of scalability). We interpreted this as good construct validity. Responsiveness of the Katz 5 item ADL list was questionable except in groups with visual and cognitive impairment [46].

A few recent articles concerned the development of the Functional Independence and Difficulty Scale (FIDS), a Japanese ADL instrument with 14 items [64–66]. This scale was validated in a Japanese population and showed good reliability (Cronbach’s *α* = 0.92; Spearman’s correlation with 6 items Katz list 0.81; test–retest correlation > 0.90; correlation with Barthel Index in healthy older adults 0.30 and frail older adults 0.80). The FIDS is not as sensitive to ceiling effects compared to the Barthel Index in healthy and in frail older people [65].

The Barthel Index consists of ten items and is predominantly used with patient populations and infrequently used for community-dwelling people. The wording of the items in the Barthel Index differed between articles (for example “Eating” or “Feeding”; “Walking” or “Mobility”). In addition, the range of the scoring was different (for example 0–100 or 0–20). Korner-Bitensky et al. [28] reported similarities in the face-to-face version and phone version > 90% (ICC 0.89). However, the phone version was unreliable in moderate and severe disabled patients. Thygese et al. [29] reported a Cronbach’s *α* of 0.82 showing a good reliability of the Barthel Index. Both Setiati et al. [30] and Wong et al. [31] reported good responsiveness of the Barthel Index. Unfortunately, validity was not sufficiently investigated, and therefore, could not be rated.

The remaining instruments listed in Table 2 (Katz 6 items, Katz 7 items, OARS, Katz unspecified, NHIS ADL and FIM phone and observation version) have less than three summarized + scores in total, and therefore, were not included here.

## Discussion

Our aim was to assess ADL instruments for use in older community-dwelling populations on psychometric properties. We identified the SMAF, 5-item Katz, FIDS and Barthel index as the four most valid and reliable ADL instruments in this field. To our knowledge, this is the first systematic review of ADL instruments utilized in community-dwelling older persons, and we regard this as a strength. After evaluating more than 6000 abstracts, and, more than a 1000 full text articles, we conclude that psychometric information is mostly not sufficiently included. Reporting of the psychometric information spanned from no information at all to very specific information, which made the evaluation a very hard effort. Additionally, different terms were used in the articles (such as convergent validity, discriminant validity, discriminative validity, etc.) and the operational definition of these terms did sometimes not match the definitions provided in Table 1. For instance, construct validity could have a broader definition as the overarching type subsuming all other types (see https://en.wikipedia.org/wiki/Construct_validity). The field of psychometric research needs more consensus about the use of these terms.

Only around 10% of the articles reported on the used ADL instrument appropriately and provided some valuable data about their reliability and validity in an older population. In total, we found 51 different ADL instruments, sometimes as remarkable as the Prayer ADL list [68]. Unexpectedly for the authors, almost no ceiling effects have been reported in the reviewed articles. It seems that this is a blind spot in research using ADL instruments in older populations. Strange, because ceiling effects lower the value of the instruments used as explained in the “Introduction” section.

It took us several years to evaluate all full text articles that we found, but it is still possible that more information about available instruments could be found in other articles or publications (see for instance our footnote on the NHIS ADL in Appendix 3). It is possible that other research was not found given the specific inclusion and exclusion criteria. Therefore, we regard this systematic review as a not all-inclusive overview of the field.

Additionally, because 60 years was set as a minimum, we excluded some interesting articles such as that of Reijneveld et al. [69]. This study among older ethnic groups in The Netherlands included a good description of the used (modified) Katz ADL items and adequate reporting on reliability and validity.

LaPlante [47] describes the developmental hierarchical model behind the Katz instruments with a reverse development in older age. Lazaridis et al. [18] checked this model in a very interesting study and found that Transfer (9.9% disability) and Incontinence (12.3% disability) have a far higher prevalence in an older population when compared to other disabilities than expected by the theory of Katz. This makes the theoretical basis for using the Katz inventory in an older population questionable.

As we chose to evaluate ADL (sub)scales and to omit combined ADL/IADL scales, we could not make any conclusions on combined lists. Perhaps this was not an optimal choice as La Plante [47] concludes that “the advantages of the IADL/ADL measure include its unbiasedness by age, greater content validity, and greater sensitivity than the ADL measure”. A combined ADL/IADL list that we encountered in our search was for instance the GARS [70]. This scale has been developed using a Mokken scale, and has good reliability and validity in an older population.

The widely used OECD disability indicator [2A] contains besides some ADL items also items on hearing, seeing and mobility. However, the textbook by McDowell summarizing properties of health measures [2A],reports this scale as with poor validity and reliability.

The Barthel Index is a well-known instrument for measuring ADL in patient populations. We found that it could also be used in older population with reasonable reliability and good responsiveness. Unfortunately, validity issues were not mentioned in the articles that we read while a review (not included in our search) by Sainsbury et al. [71] revealed problems with the reliability in people with cognitive impairments.

The six included articles on the SMAF were written by only two first authors (respectively Hebert and Desrosiers) between 1988 and 2012. As Hebert was the developer of this list, this may have been led to a bias in our review process, because some articles contained the same information. However, as all these articles were of good quality we think that we can safely recommend this instrument. Hebert [53] reveals also that the SMAF ADL could explain 57% of the variance in healthcare costs in community-dwelling older people, showing its usefulness in screening.

Frequently, we found articles concerning the OARS scale (see Table 2). Although the articles were evaluated as good quality, the findings revealed that reliability of the OARS was not very good, and validity was moderate. Doble and Fisher [23] mentioned the scale items were poorly targeted to an older population since almost half of their sample received maximal scores revealing rather low responsiveness and high-ceiling effects. They also found that continence and bathing have no good fit in their models and they have a plea for a combined measurement of ADL and IADL instead of a single ADL scale.

One of the articles concerning the NHIS ADL scale (see Table 2) was written by Jobe and Mingay [60]. They report a very interesting qualitative study revealing that respondents have a lot of problems with interpreting the questions on ADL: for example, denying problems in dressing by adjusting the clothes to be able to cope with tendonitis. Therefore, respondents clearly tend to underreport physical difficulties thereby compromising the validity of the questions (it did not measure what it was supposed to measure).

In general, as most ADL instruments use the same kind of questions with the same kind of answer categories we think that this problem not only accounts for the NHIS ADL but also for other (ADL) scales (for a good overview see Choi and Pak [72]). The problem with wording was also addressed by Rodgers and Miller [73] concerning a comparative analysis of ADL questions in surveys of older people: “… it is apparent that seemingly minor differences in the wording of questions can have large effects on the proportion of elderly respondents who report difficulty or the receipt of help with specific ADLs, the proportion who report any ADL limitation, and the average number of ADL limitations”. Together, with the earlier described problem that instruments could differ in the choice of the items used, this poses a barrier for implementation of ADL lists. This was also noticed and mentioned already in 1980 by Dunt et al. [74] who pointed to the importance of including the use of aids and assistance in the answer categories of ADL lists. Unfortunately, few instruments reviewed followed that advice (as far as we could see, the SMAF, FIDS and Katz instruments do not include the use of aids in the answer categories; the Barthel mentions the use of aids while walking and climbing stairs; the NHIS ADL questions asks if and which aids are to be used). Given the number, older people that use walkers and wheeling chairs, it is an important point that should be addressed in the future.

Instruments developed for patient populations were excluded. We encountered a review concerning ADL lists for dementia patients by Sikkes [75] that concludes “The findings indicate that improvements in and more data on psychometric properties of (I)ADL questionnaires for dementia patients are necessary to justify their use”. This gives the same impression as our own review, namely that still lot of improvement need to be made.

An older review by Law and Letts [76] in the occupational therapy area suggests the use of home-made ADL checklists should be discontinued. The FIDS [64] is a recent home-made ADL checklist, however, it was developed in a thorough manner with enough quality for recommendation. The disadvantage is that it has only been utilized in research on Japanese populations.

## Conclusion

In conclusion, only a few well-documented valid and reliable measures for ADL in populations of community-dwelling older people exist. Critical reflection in this field is necessary to avoid unnecessary modifications and the use of instruments that have not been documented to be valid and reliable. We recommend the SMAF and the FIDS in ADL screening and assessment. The Barthel Index and the 5-item list Katz inventory may be used with care concerning wording and items included.

## Electronic supplementary material

Below is the link to the electronic supplementary material.

Supplementary material 1 (DOCX 22 KB)

Supplementary material 2 (DOCX 19 KB)

Supplementary material 3 (XLSX 15 KB)
