# Supplementary material for: Activities of daily living in older community-dwelling persons: a systematic review of psychometric properties of instruments
Source: Aging Clin Exp Res. 2018 Sep 6;31(7):917–25. doi: 10.1007/s40520-018-1034-6 (PMC6589141; doi:10.1007/s40520-018-1034-6)
Supplement: Supplementary file 1 — Supplementary material 1 (DOCX 22 KB) [file 40520_2018_1034_MOESM1_ESM.docx]

**Appendix 1: Additional references**

11. Rozzini R., Frisoni GB, Ferruci L, Barbisoni P, Trabucchi M (1997) The effect of chronic diseases on physical function. Comparison between activities of daily living scales and the Physical Performance Test. Age Ageing 26(4):281-7

12. Chodosh J, Miller-Martinez D, Aneshensel CS, Wight RG, Karlamangla AS (2010) Depressive symptoms, chronic diseases, and physical disabilities as predictors of cognitive functioning trajectories in older Americans. J Am Geriatr Soc 58(12):2350-7 <https://doi.org/10.1111/j.1532-5415.2010.03171.x>

13. Beckett LA, Brock DB, Lemke JH, Mendes de Leon CF, Guralnik JM, Fillenbaum GG, et al. (1996) Analysis of change in self-reported physical function among older persons in four population studies. Am J Epidemiol 143(8):766-78

14. Wight RG, Cummings JR, Miller-Martinez D, Karlamangla AS, Seeman TE, Aneshensel CS (2008) A multilevel analysis of urban neighborhood socioeconomic disadvantage and health in late life. Soc Sci Med 66(4):862-72 <https://doi.org/10.1016/j.socscimed.2007.11.002>

15. Kai I, Ohi G, Kobayashi Y, Ishizaki T, Hisata M, Kiuchi M (1991) Quality of life: a possible health index for the elderly. Asia Pac J Public Health 5(3):221-7 <https://doi.org/10.1177/101053959100500306>

16. Stineman MG, Xie D, Pan Q, Kurichi JE, Saliba D, Streim J (2011) Activity of daily living staging, chronic health conditions, and perceived lack of home accessibility features for elderly people living in the community. J Am Geriatr Soc 59(3):454-62 <https://doi.org/10.1111/j.1532-5415.2010.03287.x>

17. Pressley JC, Patrick CH (1999) Frailty bias in comorbidity risk adjustments of community-dwelling elderly populations. J Clin Epidemiol 52(8):753-60

19. Andre MB, Dumavibhat N, Ngatu NR, Eitoku M, Hirota R, Suganuma N (2013) Mini Nutritional Assessment and functional capacity in community-dwelling elderly in Rural Luozi, Democratic Republic of Congo. Geriatric Gerontol Int 13(1):35-42 <https://doi.org/10.1111/j.1447-0594.2012.00852.x>

20. Montejo P, Montenegro M, Fernández MA, Maestú F (2012) Memory complaints in the elderly: quality of life and daily living activities. A population based study. Arch Gerontol Geriatr 54(2):298-304 <https://doi.org/10.1016/j.archger.2011.05.021>

21. Fillenbaum GG, Smyer MA (1981) The development, validity, and reliability of the OARS multidimensional functional assessment questionnaire. J Gerontol 36(4):428-34

22. Ramos LR, Simoes EJ, Albert MS (2001) Dependence in activities of daily living and cognitive impairment strongly predicted mortality in older urban residents in Brazil: A 2-year follow-up. J Am Geriatr Soc 49(9):1168-75

24. Whitelaw NA, Liang J (1991) The structure of the OARS physical health measures. Med Care 29(4):332-47

25. Thomas VS, Rockwood K, McDowell I (1998). Multidimensionality in instrumental and basic activities of daily living. J Clin Epidemiol 51(4):315-21

26. George LK, Fillenbaum GG (1985) OARS methodology: A decade of experience in geriatric assessment. JAGS 33(9):607-15

27. Puente AN, Terry DP, Faraco CC, Brown CL, Miller LS (2014) Functional impairment in mild cognitive impairment evidenced using performance-based measurement. J Geriatr Psychiatry Neurol 27(4):253-8 <https://doi.org/10.1177/0891988714532016>

28. Korner-Bitensky N, Wood-Dauphinee, S (1995) Barthel Index information elicited over the telephone: is it reliable? Am J Phys Med Rehabil 74(1):9-18

29. Thygesen E, Lindstrom TC, Saevareid HI, Engedal K (2009) The subjective health complaints inventory: A useful instrument to identify various aspects of health and ability to cope in older people? Scand J Public Health 37(7):690-6 <https://doi.org/10.1177/1403494809344104>

30. Setiati S, Harimurti K, Dewiasty E, Istanti R (2011) Predictors and scoring system for health-related quality of life in an Indonesian community-dwelling elderly population. Acta Med Indones 43(4):237-42

31. Wong DD, Wong RP, Caplan GA (2007) Self-rated health in the unwell elderly presenting to the emergency department. Emerg Med Australas 19(3):196-202 <https://doi.org/10.1111/j.1742-6723.2007.00924.x>

32. Kim YP, Kim S, Joh JY, Hwang HS (2014) Effect of interaction between dynapenic component of the European working group onsarcopenia in older people sarcopenia criteria and obesity on activities of daily living in the elderly. J Am Med Dir Assoc 15(5):371e1-e5 <https://doi.org/10.1016/j.jamda.2013.12.010>

33. Woo J, Yu R, Wong M, Yeung F, Wong M, Lum C (2015) Frailty Screening in the Community Using the FRAIL Scale. J Am Med Dir Assoc 16(5):412-9 <https://doi.org/10.1016/j.jamda.2015.01.087>

34. Yamada M, Arai H (2015) Predictive Value of Frailty Scores for Healthy Life Expectancy in Community-Dwelling Older Japanese Adults. J Am Med Dir Assoc 16(11):1002.e7-11 <https://doi.org/10.1016/j.jamda.2015.08.001>

35. Alexander NB, Guire KE, Thelen DG, Ashton-Miller JA, Schultz AB, Grunawalt JC, et al. (2000) Self-reported walking ability predicts functional mobility performance in frail older adults. J Am Geriatr Soc 48(11):1408-1413

36. Mahoney D, Tennstedt S, Friedman R, Heeren T (1999) An automated telephone system for monitoring the functional status of community-residing elders. Gerontologist 39(2):229-34

37. Magaziner J, Bassett SS, Hebel JR, Gruber-Baldini A (1996) Use of proxies to measure health and functional status in epidemiologic studies of community-dwelling women aged 65 years and older. Am J Epidemiol 143(3):283-92

38. Reyes-Ortiz CA, Kuo YF, Dinuzzo AR, Ray LA, Raji MA, Markides KS (2005) Near vision impairment predicts cognitive decline: Data from the Hispanic established populations for epidemiologic studies of the elderly. J Am Geriatr Soc 53(4):681-6 <https://doi.org/10.1111/j.1532-5415.2005.53219.x>

39. Al Snih S, Markides KS, Ottenbacher KJ, Raji MA (2004) Hand grip strength and incident ADL disability in elderly Mexican Americans over a seven-year period. Aging Clin Exp Res 16(6):481-6 <https://www.ncbi.nlm.nih.gov/pmc/articles/PMC1635471/>

40. Smith LA, Branch LG, Scherr PA, Wetle T, Evans DA, Hebert L., et al. (1990) Short-term variability of measures of physical function in older people. J Am Geriatr Soc 38(9):993-8

41. Shmotkin D, Lerner-Geva L, Cohen-Mansfield J, Blumstein T, Eyal N, Shorek A, et al. (2010) Profiles of functioning as predictors of mortality in old age: the advantage of a configurative approach. Arch Geriontol Geriatr 51(1):68-75 <https://doi.org/10.1016/j.archger.2009.07.010>

42. Covinsky KE, Palmer RM, Counsell SR, Pine ZM, Walter LC, Chren MM (2000) Functional status before hospitalization in acutely ill older adults: Validity and clinical importance of retrospective reports. JAGS 48(2):164-9

43. Chou KL, Chi I (2005) Prevalence and correlates of depression in Chinese oldest-old. Int J Geriatr Psychiatry 20(1):41-50 <https://doi.org/10.1002/gps.1246>

44. Seematter-Bagnoud L, Santos-Eggimann B, Rochat S, Martin E, Karmaniola A, Aminian K, et al. (2010) Vulnerability in high-functioning persons aged 65 to 70 years: The importance of the fear factor. Aging Clin Exp Res 22(3):212-8 <https://doi.org/10.3275/6705>

45. Wolinsky FD, Coe RM, Miller DK, Prendergast JM (1984) Measurement of the global and functional dimensions of health status in the elderly. J Gerontol 39(1):88-92

46. Whitson HE, Cousins SW, Burchett BM, Hybels CF, Pieper CF, Cohen HJ (2007) The combined effect of visual impairment and cognitive impairment on disability in older people. JAGS 55(6):885-891 <https://doi.org/10.1111/j.1532-5415.2007.01093.x>

48. Kosloski K, Datwyler MM, Montgomery RJV (1994) Evaluating retrospective measures in gerontological research. Res Aging 16(4):389-400 <http://journals.sagepub.com.vu-nl.idm.oclc.org/doi/abs/10.1177/0164027594164003>

49. Hebert R, Bravo G, Korner-Bitensky N, Voyer L (1996) Predictive validity of a postal questionnaire for screening community-dwelling elderly individuals at risk of functional decline. Age Ageing 25(2):159-67

50. Hebert R, Carrier R, Bilodeau A (1988) The functional autonomy measurement system (SMAF): Description and validation of an instrument for the measurement of handicaps. Age Ageing 17(5):293-302

51. Hebert R, Raiche M, Gueye NR (2012) Survey disability questionnaire does not generate valid accurate data compared to clinical assessment on an older population. Arch Gerontol Geriatr 54(2):e57-e62 <https://doi.org/10.1016/j.archger.2011.06.021>

52. Desrosiers J, Noreau L, Robichaud L, Fougeyrollas P, Rochette A, Viscogliosi C (2004) Validity of the Assessment of Life Habits in older adults. J Rehabil Med 36(4):177-82 <https://doi.org/10.1080/16501970410027485>

53. Desrosiers J, Bravo G, Hébert R, Dubuc N (1995) Reliability of the revised functional autonomy measurement system (SMAF) for epidemiological research. Age Ageing 24(5):402-6

54. Hebert R, Spiegelhalter DJ, Brayne C (1997) Setting the minimal metrically detectable change on disability rating scales. Arch Phys Med Rehabil 78(12):1305-8

55. Rodgers W, Miller, B (1997) A comparative analysis of ADL questions in surveys of older people. J Gerontol Ser B Psychol Sci Soc Sci 52(SPEC. ISS.):21-36

56. Cullen JS, Grayson DA, Jorm AF (1996) Clinical diagnoses and disability in cognitively impaired older persons. Int J Geriatr Psychiatr 11(5):411-22

57. Weiner DK, Duncan PW, Chandler J, Studenski SA (1992) Functional reach: A marker of physical frailty. J Am Geriatr Soc 40(3):203-7

58. Crawford SL, Jette AM, Tennstedt SL (1997) Test-retest reliability of self-reported disability measures in older adults. J Am Geriatr Soc 45(3):338-341

59. Schonberg MA, Davis RB, McCarthy EP, Marcantonio ER (2009) Index to predict 5-year mortality of community-dwelling adults aged 65 and older using data from the National Health Interview Survey. J Gen Intern Med 24(10):1115-22 <https://doi.org/10.1007/s11606-009-1073-y>

61. Petrella RJ, Overend T, Chesworth B (2002) FIM after hip fracture: is telephone administration valid and sensitive to change? Am J Phys Med Rehabil 81(9):639-44 <https://doi.org/10.1097/01.CCM.0000026916.24522.BD>

61. Cotter EM, Burgia LD, Stevens AB, Roth DL, Gitlin LN (2002) Correspondence of the Functional Independence Measure (FIM) self-care subscale with real-time observations of dementia patients' ADL performance in the home. Clin Rehabil 16(1):36-45 <https://doi.org/10.1191/0269215502cr465oa>

63. Chang WC, Slaughter S, Cartwright D, Chan C (1997) Evaluating the FONE FIM: Part I. Construct validity. J Outcome Meas 1(3):192-218

64. Saito T, Izawa KP, Omori Y, Watanabe S (2016) Functional Independence and Difficulty Scale: Instrument development and validity evaluation. Geriatr Gerontol Int 16(10):1127-1137 <https://doi.org/10.1111/ggi.12605>

65. Saito T, Izawa KP, Matsui N, Arai K, Ando M, Morimoto K, et al (2017) Comparison of the measurement properties of the Functional Independence and Difficulty Scale with the Barthel Index in community-dwelling elderly people in Japan. Aging Clin Exp Res 29(2):273-281 <https://doi.org/10.1007/s40520-016-0558-x>

66. Saito T, Izawa KP, Watanabe S (2017) The relative and absolute reliability of the Functional Independence and Difficulty Scale in community-dwelling frail elderly Japanese people using long-term care insurance services. Aging Clin Exp Res 29(3):549-556 <https://doi.org/10.1007/s40520-016-0577-7>
