# Supplementary material for: Activities of daily living in older community-dwelling persons: a systematic review of psychometric properties of instruments
Source: Aging Clin Exp Res. 2018 Sep 6;31(7):917–25. doi: 10.1007/s40520-018-1034-6 (PMC6589141; doi:10.1007/s40520-018-1034-6)
Supplement: Supplementary file 2 — Supplementary material 2 (DOCX 19 KB) [file 40520_2018_1034_MOESM2_ESM.docx]

**Appendix 2: Search strategies**

**Embase**

('daily life activity'/exp OR 'ADL disability'/de

OR

'functional status'/de OR 'functional

assessment'/de OR

(((

daily OR living* OR everyday OR 'every day' OR 'real world'

)

NEAR/3

(activit* OR action* OR tasks OR functioning OR dysfunction*

OR competence* OR

incompetence*)) OR (function

al

NE

XT/1 (abilit* OR disabilit* OR independ* OR depend*

OR impair* OR perform* OR deficit* OR skill* OR limit* OR decline* OR

status OR living

OR capacit* OR mobilit* OR competenc* OR assess*)

)

OR (independence NEAR/3 (loss OR

losing)) OR (perform* NEAR/3 limitat*) OR

((

basic OR personal OR self) NEXT/2 (care OR

help)) OR (independ* NEAR/3 level*) OR (Limitat* NEAR/3 Activit*) OR

(physic*

NEAR/3 function*) OR ADL OR ADL? OR BADL OR BADL? OR IADL OR IAD

L?):ab,ti

) AND (questionnaire/exp OR (Questionnaire* OR Checklist* OR (Surve

y NEAR/2

instrument*) OR (Self NEAR/3 (report* OR assess*)) OR Measuremen

t* OR

Measures):ab,ti) AND (aged/de OR 'frail elderly'/de OR 'very elder

ly'/de OR (((Older OR old)

NEXT/1 (adult* OR person* OR people OR individual* OR women OR men)) OR

aging OR

ageing OR elderly OR (aged NOT ((boy* OR girl* OR child*) NEXT/1 aged)

OR 'school

aged') OR senior* OR centenarian* OR nonagenarian* OR octagena

rian* OR septagenarian*

OR ((later OR late) NEAR/2 life)

OR geriatr* ):ab,ti) AND ('validation study'/de OR

valid*:ab,ti OR reproducibility/de OR reproducib* OR reliability/exp OR re

liab*:ab,ti OR

repeatab*:ab,ti OR 'correlation coefficient'/de OR 'intracla

ss correlation':ab,ti OR 'internal

consistency'/de OR consistenc*:ab,ti OR responsive*:ab,ti OR agree

ment:ab,ti OR ' factorial

analysis'/de OR 'factor analys?s':ab,ti OR 'factor structure':a

b,ti OR dimension*:ab,ti OR

'sensitivity and specificity'/de OR sensitiv*:ab,ti OR clinime

tr*:ab,ti OR psychometr*:ab,ti

OR psychometry/exp OR (measur* NEAR/3 propert*):ab,ti OR ((smallest

OR minimal*)

NEXT/1 (important OR detectable OR real)):ab,ti OR variation:ab,t

i OR precision:ab,ti OR

imprecision:ab,ti OR ((interobserver OR 'inter observer'

OR interrater OR 'inter rater' OR

intraobserver OR 'intra observer' OR intrarater OR 'in

tra rater') NEXT/1 variability):ab,ti OR

stabilit*:ab,ti OR error*:ab,ti OR prediction:ab,ti)

#1 NOT ((hospital* OR patient* OR inpatient* OR (severe* NEAR/3 ill*)

OR ((nursing OR

elderly OR aged

)

NEAR/3 home*)):ab,ti OR (care NEXT/1 home*):ab,ti OR 'clinical

practice':ab,ti OR hospital/exp OR patient/exp OR ('physical di

sease'/exp NOT disability/exp)

OR 'mental disease'/exp OR 'home for the aged'/de OR 'nursing

home'/de OR 'therapy'/exp)

OR

#1 AND ((

((

Community OR independ*) NEAR/4 (dwelling OR dwellers OR residing OR

residents OR based OR live OR lived OR living OR setting* OR sample

))

OR

communitydwell* OR (residential NEXT/1 setting*) OR healthy OR (nati

onal* NEAR/6

(survey OR sample)) OR noninstitutionali?ed OR 'non instituti

onali?ed' OR nonhospitali?ed

OR 'non hospitali?ed' ):ab,ti OR (home* NOT ((care NEXT/1 hom

e*) OR (home* NEAR/3

(aged* OR elderly OR nursing)):ab,ti OR 'home for the aged'/de OR 'n

ursing home'/de)) OR

'independent living'/de OR 'community living'/de OR 'home environment

'/de OR 'community

sample'/de OR (('cohort analysis'/de OR cohort*) NOT ((hospit

al* OR patient*):ab,ti OR

hospital/exp OR patient/exp )) OR 'normal human'/de)

**OvidSP:**

**Medline**

**PsycINFO**

**Amed**

(exp Activities of Daily Living/ OR (((daily OR living* OR everyday

OR every day OR real

world) ADJ3 (activit* OR action* OR task* OR functioning OR dysfunct

ion* OR

competence* OR incompetence*)) OR (functional ADJ (abilit* OR d

isabilit* OR independ*

OR depend* OR impair* OR perform* OR deficit* OR skill* OR limit* OR decli

ne* OR

status OR living OR capacit* OR mobilit* OR competenc* OR assess*)

) OR (independence

ADJ3 (loss OR losing)) OR ((basic OR personal OR self) AD

J2 (care OR help)) OR

(independ* ADJ3 level*) OR (Limitat* ADJ3 Activit*) OR ADL? OR BADL?

OR

IADL?).tw,ot,kw. ) AND (questionnaires/ OR (Questionnaire* OR Chec

klist* OR (Survey

ADJ2 instrument*) OR (Self ADJ3 (report* OR assess*)) OR Mea

surement* OR

Measures).tw,ot,kw.) AND (exp aged/ OR (((Older OR old) ADJ (adul

t* OR person* OR

people OR individual* OR women OR men)) OR aging OR ageing OR elder

ly OR (aged

NOT ((boy* OR girl* OR child) ADJ aged) OR school aged) OR senior

* OR centenarian*

OR nonagenarian* OR octagenarian* OR septagenarian* OR ((later

OR late) ADJ2 life) OR

geriatr*).tw,ot,kw.) AND (Validation Studies as Topic/ OR Validat

ion Studies.pt. OR

valid*.tw,ot,kw. OR Reproducibility of Results/ OR reproducib*.tw,ot,kw.

OR

reliab*.tw,ot,kw. OR repeatab*.tw,ot,kw. OR intraclass correlat

ion.tw,ot,kw. OR

consistenc*.tw,ot,kw. OR responsive*.tw,ot,kw. OR agreement.tw,

ot,kw. OR (factor* adj

(analys?s OR structure)).tw,ot,kw. OR dimension*.tw,ot,kw. OR "sensi

tivity and specificity"

/

OR sensitiv*.tw,ot,kw. OR clinimetr*.tw,ot,kw. OR psychometr*.tw,ot,kw. OR

Psychometrics/ OR (measur* ADJ3 propert*).tw,ot,kw. OR ((smallest OR

minimal*) ADJ

(important OR detectable OR real)).tw,ot,kw. OR variation.tw,ot,

kw. OR precision.tw,ot,kw.

OR imprecision.tw,ot,kw. OR ((interobserver OR inter observe

r OR interrater OR inter rater

OR intraobserver OR intra observer OR intrarater OR int

ra rater) ADJ variability).tw,ot,kw.

OR stabilit*.tw,ot,kw. OR error*.tw,ot,kw. OR prediction.tw,ot,kw.)

1 NOT ((hospital* OR patient* OR inpatient* OR (severe* ADJ3 ill*)

OR ((nursing OR

elderly OR aged) ADJ3 home*)).ab,ti. OR care home*.ab,ti. OR

clinical practice.ab,ti. OR

exp hospitals/ OR exp patients/ OR exp Mental Disorders/ OR ho

mes for the aged/ OR

nursing homes/ OR exp Therapeutics/ OR therapy.xs.)

OR

1 AND ((((Community OR home OR independ*) ADJ4 (dwelling OR dwellers

OR residing

OR residents OR based OR live OR lived OR living OR setting* OR sampl

e)) OR

communitydwell* OR residential setting* OR Rural* OR healthy OR n

oninstitutionali?ed OR

non institutionali?ed OR nonhospitali?ed OR non hospitali?

ed ).tw,kw,ot. OR (home* NOT

(care home* OR (home* ADJ3 (aged OR elderly OR nursing)).tw,kw,ot.

OR homes for the

aged/ OR nursing homes/)) OR independent living/ OR Residence Char

acteristics/ OR Rural

Population/ OR (exp Cohort Studies/ NOT ((hospital* OR patient*)

.ab,ti. OR exp hospitals/

OR exp patients/ )) OR exp Population Groups/)

**Cinahl**

SU ((((daily OR living* OR everyday OR every day OR real world) N3

(activit* OR action*

OR functioning OR dysfunction* OR competence* OR incompetenc

e*)) OR (functional N1

(abilit* OR disabilit* OR independ* OR depend* OR impair* OR perform* OR de

ficit* OR

skill* OR limit* OR decline* OR status OR living OR capacit* OR mobilit* OR

competenc*

OR assess*)) OR (independence N3 (loss OR losing)) OR ((basi

c OR personal OR self) N2

(care OR help)) OR (independ* N3 level*) OR (Limitat* N3 Activit*)

OR (physic* N3

function*) OR ADL* OR BADL* OR IADL*) ) AND ((Questionnaire* OR C

hecklist* OR

(Survey N2 instrument*) OR (Self N3 (report* OR assess*)) OR Mea

surement* OR

Measures)) AND ((((Older OR old) N1 (adult* OR person* OR people OR i

ndividual* OR

women OR men)) OR aging OR ageing OR elderly OR (aged NOT ((boy* OR

girl* OR

child) N1 aged OR school aged)) OR senior* OR centenarian* OR non

agenarian* OR

octagenarian* OR septagenarian* OR ((later OR late) N2 life)

OR geriatr*)) AND (valid*

OR reproducib* OR reliab* OR repeatab* OR intraclass correlatio

n OR consistenc* OR

responsive* OR agreement OR (factor* N1 (analys*s OR structure)

) OR dimension* OR

sensitiv* OR clinimetr* OR psychometr* OR (measur* N3 propert*) OR

((smallest OR

minimal*) N1 (important OR detectable OR real)) OR variation

OR precision OR

imprecision OR ((interobserver OR inter observer OR inter

rater OR inter rater OR

intraobserver OR intra observer OR intrarater OR intra

rater) N1 variability) OR stabilit* OR

error* OR prediction)

AND

SU (((Community OR independ*) N4 (dwelling OR dwellers OR

residing OR residents OR based OR live OR lived OR living OR setting*

OR sample)) OR

communitydwell* OR residential setting* OR healthy OR (national*

N6 (survey OR sample))

OR noninstitutionali?ed OR non institutionali?ed OR nonhospitali

?ed OR non hospitali?ed

OR (home* NOT (care home* OR (home* N3 (aged* OR elderly OR nursing)

) ) ))
